# Events with Different Emotional Valence Affect the Eye’s Lacrimal Caruncle Temperature Changes in Sheep

**DOI:** 10.3390/ani14010050

**Published:** 2023-12-22

**Authors:** Marta Comin, Elie Atallah, Matteo Chincarini, Silvia Michela Mazzola, Elisabetta Canali, Michela Minero, Bruno Cozzi, Emanuela Rossi, Giorgio Vignola, Emanuela Dalla Costa

**Affiliations:** 1 Dipartimento di Medicina Veterinaria e Scienze Animali, Università degli Studi di Milano, 26900 Lodi, Italy; marta.comin@unimi.it (M.C.); elie.atallah@unimi.it (E.A.); silvia.mazzola@unimi.it (S.M.M.); elisabetta.canali@unimi.it (E.C.); michela.minero@unimi.it (M.M.); 2 Facoltà di Medicina Veterinaria, Università degli Studi di Teramo, 64100 Teramo, Italy; m.chincarini@unite.it (M.C.); g.vignola@unite.it (G.V.); 3 Dipartimento di Biomedicina Comparata e Alimentazione, Università degli Studi di Padova, 35131 Padova, Italy; bruno.cozzi@unipd.it; 4 Istituto Zooprofilattico Sperimentale dell’Abruzzo e del Molise G. Caporale, 64100 Teramo, Italy; e.rossi@izs.it

**Keywords:** classical conditioning, emotional state, fear, infrared thermography, non-invasive measure, sheep welfare

## Abstract

**Simple Summary:**

We explored the application of infrared thermography (IRT) as a non-invasive method to measure the eye’s lacrimal caruncle temperature in sheep, specifically focusing on its potential to detect negative emotions such as fear. Fourteen ewes, divided into two study groups according to the exposed emotional (positive *n* = 7 and negative *n* = 7), were subjected to classical conditioning associating a neutral auditory stimulus with either a positive event (food reward) or a negative event (opening an umbrella). Then, lacrimal caruncle temperatures using IRT, behavioral observation of ear postures, and saliva samples were collected to assess cortisol levels at rest and after treatment. The results reveal a significant increase in lacrimal caruncle temperature post-treatment only in the negative group (*p* = 0.017), indicating a potential association between IRT-measured eye temperature and negative emotional states in sheep. Additionally, behavioral observations align with the learned association between the neutral stimulus and events of different emotional valence. As for cortisol, despite not being significant, its levels increased for both groups post-treatment. The study concludes that IRT proves valuable in non-invasively evaluating the physiological impact of positive and negative events on sheep.

**Abstract:**

Infrared thermography (IRT) has been recently applied to measure lacrimal caruncle temperature non-invasively since this region is related to the sympathetic response, and it seems a promising technique that is able to infer negative emotions in sheep (e.g., fear). However, the scientific literature so far is limited in understanding whether a caruncle’s temperature changes also in response to positive emotional states in sheep. Through classical conditioning, we aimed to assess how a positive or a negative event affects the physiological (lacrimal caruncle temperature measured with IRT and cortisol levels) and behavioral responses of sheep (ear position). Fourteen ewes from the same flock were randomly assigned to two treatment groups: positive (*n* = 7) and negative (*n* = 7). Each group was then trained through classical conditioning to associate a neutral auditory (ring bell) stimulus to an oncoming event: for the positive group, the presence of a food reward (maize grains), while for the negative one, the opening of an umbrella. After three weeks of training, before (at rest) and after (post-treatment), lacrimal caruncle temperature was non-invasively measured via IRT, and saliva samples were gently collected to measure cortisol levels. During treatment, sheep behavior was videorecorded and then analyzed using a focal animal sampling technique. At rest, the eye’s lacrimal caruncle temperature was similar in both groups, while post-treatment, a significant increase was shown only in the negative group (*t*-test; *p* = 0.017). In the anticipation phase, sheep in the positive group kept their ears forward longer compared to those in the negative one (Mann–Whitney; *p* < 0.014), 8.3 ± 2.1 s and 5.2 ± 4.2 s, respectively. The behavioral response observed reflects a learnt association between a neutral stimulus and events with different emotional valence. Cortisol concentration slightly increased in both groups post-treatment. Our results confirm that IRT is a non-invasive technique that can be useful when applied to assess how positive and negative events may affect the physiological response in sheep.

## 1. Introduction

The recognition that animals are sentient beings, capable of feeling emotions, is a driving force to finding non-invasive indicators for recognizing animals’ emotional state, which is an important aspect of their welfare [1,2]. Emotions can be defined as a short-lived and intense affective response to an event characterized by universal facial expressions, phylogenetic continuity of expressed behaviors, physiological activation patterns, and unconscious stimuli evaluation mechanisms [3,4]. From an evolutionary standpoint, emotions are processes that might have evolved from basic mechanisms aimed at either directing other physiological programs or solving adaptive problems faced by a species over time [5], with the final goal of giving the animals the ability to avoid harm/punishment or to seek valuable resources/reward [6,7]. The basic emotions are found throughout all mammals and birds [8,9], and may be categorized into two broad groups, based on their valence, as negative or positive emotions. Negative emotions, like fear and anger, hold a pivotal role in ensuring survival by triggering specific responses aimed at avoiding threats or dealing with stressors through the activation of the sympathetic nervous system [10,11,12]. On the other hand, positive emotions promote social bonding by activating the parasympathetic nervous system [13,14].

Determining the precise feelings of any individual farm animal is challenging due to their inability to verbally express emotions [15]. Additionally, a single emotional expression measurement, whether derived from facial features or physiological functions, is insufficient to accurately show the farm animal’s emotional changes [16]. The study of animal emotions is still a challenge as there is no gold standard measure that can be reliably used to infer the affective state of animals, meaning that it is not possible to determine the emotion the animal is feeling by using a single indicator or measure, but a multimodal approach is still preferred [16,17,18]. The ability to non-invasively measure emotions plays a central role in animal welfare assessment as it provides valuable information regarding the outcomes of interventions aimed at improving the welfare of the animals [19].

It is well-known that emotions are linked to specific physiological changes within the body [20]: for example, plasma glucocorticoid levels can rise in reaction to acute negative stimuli [21] or in anticipation of a positive situation [22]. The measurement of cortisol may yield confusing results; its detection in high levels in farm animals may enhance performance by affecting immune and endocrine pathways [23], and may have positive effects on traits related to robustness and adaptation, such as newborn survival, resistance to bacteria and parasites, and tolerance to heat stress [24]. Moreover, the limitations of cortisol as an invasive approach [25] suggest exploring less invasive alternatives. Furthermore, emotions may influence body temperature, as they are reactions to events, whether internal or external, which hold significant importance for the organism, and this can manifest as emotional fever or stress-induced hyperthermia [26]. Hence, identifying these changes leads to the possibility to infer negative or positive emotions in animals. Travain and Valsecchi [26] in their review reported the latest studies in which infrared thermography (IRT) has been applied to measure the emotional reactions of domestic animals with a focus on stress, fear, and negative situations [26]. Moreover, they reported that the eye is a particularly suitable zone due to its abundant capillary networks, which rapidly respond to variations in blood flow present on the small areas around the rear edge of the eyelid and the *caruncula lacrimalis* [27,28,29,30]. In sheep, Cannas and colleagues [19] showed that IRT seems to be a promising technique for inferring stress and fear in sheep. The physiological reaction (change in eye temperature) was measured in ewes exposed to handling and to a human–animal test, and the findings indicated that the stressful situation increased the lacrimal caruncle temperature [19]. The possible explanation for this is that the perception of stressors such as fear or anxiety can induce an increase in heart rate and blood pressure due to increased sympathetic tone [31]. This increases body temperature, referred to as stress hyperthermia [31]. Other studies have related the surface thermal response in companion animals exposed to social isolation [32,33,34]. However, it has not been possible to establish a clear relationship between this thermal response and its meaning as an emotional state, something that could be complemented with behavioral indicators.

Indeed, considering the behavioral components of the emotion changes in ear posture might be a reliable and non-invasive tool for assessing emotional valence in sheep [20,35,36,37,38]. While most research has shown an increase in ocular temperature after a negative stimulus, some studies have shown an immediate and transient reduction [30]. Reefmann and colleagues [35] found that a negative experience (e.g., social isolation) leads to the highest number of ear posture changes and forward and asymmetric ear postures. While Boissy and colleagues [37] found a correlation between ear postures in sheep and distinct emotional responses. The backward posture is associated with fear in unfamiliar and uncontrollable unpleasant situations, the raised ear posture indicates anger in response to controllable unpleasant situations, and the asymmetric ear posture expresses surprise in sudden situations and startles responses. These identified ear postures could serve as specific emotional indicators in sheep. Furthermore, Vogeli and colleagues [39] highlighted that the duration of the ear postures is also instructive, not only the frequency with which they were shown, because different ear postures are likely to occur for a different amount of time each time they are held.

To date, there is no current scientific literature available to explore whether eye temperature undergoes changes in response to positive emotional states in sheep, such as the presence of a food reward. We hypothesize that eye temperature may vary in response to positive events, and ear posture may be correlated with physiological parameters such as cortisol and IRT, providing valuable indicators for investigating the emotional states of the sheep. We hypothesize that eye temperature may vary in response to different valence emotional events, and ear posture may be correlated with physiological measures such as cortisol and IRT, providing valuable indicators for investigating the emotional states of the sheep. The aim of this study was to evaluate, through classical conditioning, how positive or negative events influence the physiological responses (lacrimal caruncle temperature measured with IRT and cortisol levels) and behavioral reaction (i.e., ear position) in sheep.

## 2. Materials and Methods

### 2.1. Ethical Statement

Data collection on animals was carried out in accordance with relevant guidelines and regulations, and all experimental protocols were approved by the national ethical commission (Ministero della Salute, authorization n° 457/2016-PR, 919/2017-PR). If any sheep was deemed to be in greater-than-mild stress (assessed live by an independent veterinarian), then it was immediately removed from the study.

### 2.2. Animals, Housing, and Husbandry

**Figure 1:**
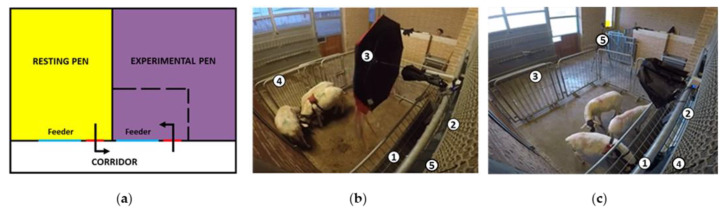
(**a**) Schematic representation of the resting and experimental pen; (**b**) experimental pen for negative group (1: feeder; 2: hidden car horn and wall covered with black plastic sheets to prevent the operator from being seen by the animals; 3: umbrella; 4: fence; 5: camera); (**c**) experimental pen for positive group (1: feeder; 2: hidden doorbell and wall covered with black plastic sheets to prevent the operator from being seen by the animals; 3: fence; 4 and 5: cameras).

This study was conducted at the experimental farm of Istituto Zooprofilattico Sperimentale dell’Abruzzo e del Molise (Italy) during the month of June in 2019. Fourteen 1-year-old Sarda ewes, not gestating nor lactating and clinically healthy, were selected and housed together in a 45 m^2^ resting pen with straw provided for bedding. In the resting pen, ewes had free access to water, and they were fed with hay twice a day (8 a.m. and 6 p.m.). Their diet was supplemented with a commercial concentrate (Mangimi Ariston Srl, Teramo, Italy; 250–300 g/sheep). The resting pen was connected to the experimental pen by a corridor 2 m long and delimited with gates specially placed to direct the path of the animals from one pen to the other (Figure 1).

The experimental pen was equipped with a 2 m long feeder, which was fixed in such a way as to remain constantly accessible by both the animals inside the box and the operators from outside. The door was covered on its outer side with black plastic sheets to prevent the operators from being seen by the sheep.

### 2.3. Animal and Study Design

Ewes were randomly assigned to two treatment groups: positive (*n* = 7) and negative (*n* = 7). Each group was trained through classical conditioning to associate a neutral auditory stimulus (doorbell and car horn for the positive and negative group, respectively) to an oncoming event: for the positive group, the presence of a food reward (maize grains), while for the negative one, the automatic opening of an umbrella covered with colored wires to make it as scary as possible for the animals.

#### Training Strategy Workflow

The training of ewes, lasting three weeks, comprised two different aspects: habituation to the experimental set up and classical conditioning of a neutral sound. To prevent animals from hearing the neutral sound when they were not in the experimental pen, the rest of the flock, not involved in the training, was kept outside in a pasture far from the experimental pen. Sheep were gradually habituated to be split from their flock in small groups of three and moved to the experimental pen (2 × 2 m^2^), where they were socially and visually isolated from the rest of the flock. To avoid social isolation stress, within each group, two “accompanying sheep” were selected. The accompanying sheep (always the same two individuals for each group) performed the training together with each individual experimental sheep, but they were not included in the data collection. The training of ewes took place daily in the afternoon at the same time between 14 and 18 and it was run by two operators. During the habituation phase (5 consecutive days), ewes entered the experimental pen and remained there for 30 min, receiving a standard food reward (i.e., 100 g of maize grains) in a bucket. The conditioning phase started after the habituation, and it was divided into two different steps. In the first step (8 consecutive days), sheep learned to associate a neutral sound lasting five seconds to an oncoming event. Through classic Pavlovian conditioning, animals of the positive group associated the sound, performed by an operator located in the corridor, with the release of maize grains in the feeder, while sheep of the negative group associated the sound with a sudden opening of the umbrella by an operator located in the corridor. Each training day, sheep underwent 10 conditioning trials. As a second step (8 consecutive days), the time between the sound and the oncoming event was gradually increased to 10 s. Ten seconds was selected as a good time interval to evaluate anticipatory behavior in sheep. All sheep were conditioned at the end of 3 weeks by ensuring that after the sound, they moved toward the feeder (positive group) or ran to a corner (negative group).

### 2.4. Data Collection and Analysis

The following table reports the different phases for each treatment group (Table 1).

#### 2.4.1. Lacrimal Caruncle Infrared Thermography

Data collection took place on the last day of the conditioning phase. The temperature (°C) of the lacrimal caruncle of each sheep was measured in the resting and in the experimental pen after completing the 10 training trials of the day after the end of the last trial consumption phase (positive group) and sheep reaction (negative group) by using an infrared camera (NEC Avio TVS500; Nippon Avionics Co., Ltd., Tokyo, Japan) with a standard optic system (accuracy ± 2 °C and resolution 320 × 240 px). Room temperature and humidity in both resting and experimental pens were automatically controlled and stable (minimum = 19.30 °C, maximum = 20.43 °C; and mean = 19.92 °C). To optimize the accuracy of the thermographic image, the same image of a Lambert surface was taken to define the radiance emission and to nullify the effect of surface reflections on the tested animals before every work session [40]. Lacrimal caruncle was selected as the target area because it has a temperature that is unaffected by the presence of hair [41,42]. Pictures (3 for each eye, left and right, taken with approximately 10 s between them) were collected from the same location, on a side with an approximate angle of 90° and from a distance range of 0.5 m from the animal. Grayess IRT Analyzer 6.0 software (Bradenton, FL, USA) was used to determine the temperature of the lacrimal caruncle, and the maximum temperature (°C) inside a circular region traced around the area was measured [43]. The emissivity values employed in the analyses were those measured for high emissivity objects (0.97 and 0.98). An example of a picture is shown in Figure 3a.

#### 2.4.2. Ear Posture

**Figure 2:**
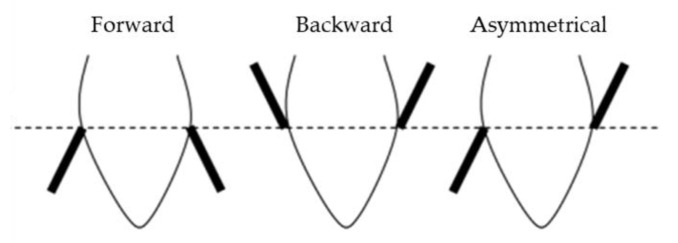
Position of the ears in relation to the frontal plane of the head (modified from Boissy et al., 2011 [37]).

Videos were then analyzed with a focal animal continuous recording method using the software Solomon Coder beta 17.03.22. The durations of different ear postures in the frontal plane (pointing forward, backward, or asymmetrical), as described by Boissy and colleagues [37] (Figure 2), were evaluated during the baseline (10 s) and anticipation phase (10 s) of the oncoming event by an independent observer not aware of the aim of the study. If the ears’ posture is oriented forward, it means that the top of the ear is in front of the frontal plane, and, on the contrary, if pointing backward, the top of the ear is behind the frontal plane. Lastly, if the two ears differ in their position with respect to the frontal plane, they are evaluated as asymmetrical.

#### 2.4.3. Cortisol

For the assessment of cortisol concentrations, saliva specimens were obtained from each sheep with a commitment to ensuring minimal distress during the procedure (sheep were previously habituated to this manipulation). SalivaBio Children’s Swabs (Salimetrics^®^, Carlsbad, CA, USA) were utilized to collect samples in both the resting pen and post-treatment, immediately following the acquisition of thermographic images. The swab, introduced into the sheep’s oral cavity for approximately 1 min, was administered with gentle restraint, allowing the animal to remain in proximity to the flock without isolation. Subsequent to sampling, the swab was deposited in the designated device tube, sealed with a plastic stopper to avert evaporation, and promptly placed in an ice container. Within an hour, the samples were frozen at −20 °C, maintaining this temperature until analysis. During analysis, the samples were thawed at room temperature, followed by centrifugation (3500 rpm for 15 min at 4 °C) in accordance with the salivary sample protocol provided by the kit manufacturer. A commercially available multispecies cortisol enzyme-linked immunosorbent assay (ELISA) kit (Enzo Life Sciences, Farmingdale, NY, USA), validated through established protocols [44], was employed for analysis. Duplicate aliquots (100 μL) of the samples were placed in wells, and absorbance was measured at a wavelength of 405 nm using a microplate reader (Multiskan EX, LabSystem, Thermo Fisher Scientific, Milan, Italy). To assess the accuracy of our samples, a recovery test was implemented, yielding a mean recovery of 109.1% ± 8.4. The intra- and inter-assay coefficients of variation averaged 3.9 and 7.8%, respectively. The assay sensitivity was determined to be 56.72 pg/mL (range 156–10,000 pg/mL). Importantly, the laboratory researcher conducting the analysis remained blinded to the study hypotheses and conditions.

### 2.5. Statistical Analysis

Data were entered into Microsoft Excel (Microsoft Corporation, 2010, Washington, DC, USA) before being analyzed with SPSS statistical package (SPSS Statistic 28, IBM, Armonk, NY, USA). The descriptive statistics (mean and standard deviations) were calculated for each of the studied variables. The data were tested for normality and homogeneity of variance using Kolmogorov–Smirnov and Levene’s tests, respectively. For thermographic data, *t*-tests were used to compare data at rest and post-treatment in both groups. Cortisol levels and ear posture data were not normally distributed; therefore, a non-parametric Mann–Whitney U test was used to identify differences between treatment groups and the Wilcoxon test was used to identify possible differences in cortisol levels at rest and post-treatment in both groups. Differences were considered statistically significant if *p* ≤ 0.05.

## 3. Results

**Figure 3:**
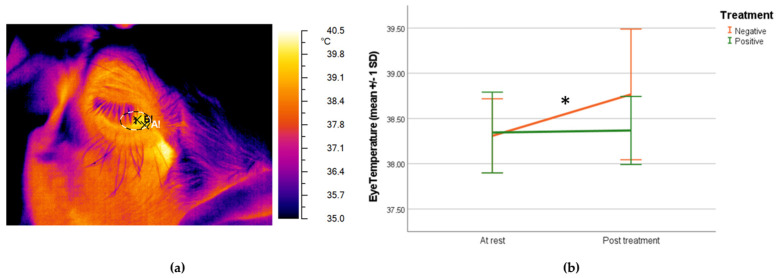
(**a**) Thermographic infrared image of the sheep’s eye with the lacrimal caruncle as the target area; (**b**) graph showing mean (±1 SD) lacrimal caruncle temperature (°C) at rest and after the treatment for both negative and positive groups (paired samples *t*-test; * *p* < 0.05).

The lacrimal caruncle’s temperature at rest was 38.31 ± 0.41 °C in the negative group and 38.35 ± 0.45 °C in the positive group with no significant differences (independent *t* test; *p =* 0.802) between the groups. After treatment, the sheep in the negative group showed a significant increase (paired samples *t*-test; *p* = 0.017) in lacrimal caruncle temperature (38.77 ± 0.72 °C), while in the positive group eye temperature remained unchanged (38.37 ± 0.33 °C), as shown in Figure 3b.

**Figure 4:**
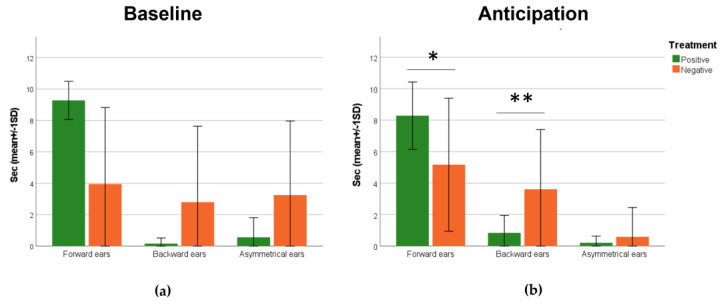
Graphs reporting the mean (±SD) duration in seconds of different ear positions of the negative of positive treatment groups during (**a**) baseline and (**b**) anticipation phases (Mann–Whitney U test; * *p* < 0.05; ** *p* < 0.01).

In the baseline phase, there are no significant differences in ear positions between both the positive and negative treatment groups (Figure 4a), while during the anticipation phase, there were statistically significant differences in ear posture between the two treatment groups (Figure 4b). Ewes in the positive group kept their ears forward longer (Mann–Whitney U test; *p* < 0.014) and backward for less seconds (Mann–Whitney U test; *p* < 0.008).

The cortisol concentration at rest was 0.43 ± 0.07 pg/mL in the negative group and 0.41 ± 0.12 pg/mL in the positive group with no significant differences (Mann–Whitney U test; *p* > 0.05) between the groups. After treatment, the sheep in both groups showed an increase in cortisol levels, although not significant (Wilcoxon test; *p* > 0.05), and the negative group’s level was 0.67 ± 0.39 pg/mL, while for the positive group, it was 0.59 ± 0.39 pg/mL.

## 4. Discussion

The study aimed at evaluating, through a classical conditioning, how positive or negative events influence the physiological responses and behavioral reaction in sheep. This was achieved by evaluating ear position and measuring eye temperature using infrared thermography and salivary cortisol levels in two groups of sheep conditioned to two different valence stimuli. The findings indicated that, post-treatment, there was a significant increase in the temperature of the lacrimal caruncle only in the negative treatment group, as opposed to prior to its commencement with the sheep in the resting box. Previous studies have provided evidence indicating that the temperature of the lacrimal caruncle in sheep is elevated during short stressful events such as restraint [19] and shearing [45]. Furthermore, several studies have also demonstrated that castration [46], transport [47], and forced lateralization test in cattle [48], trimming [49] and noseband tightening [50] in horses, and veterinary visits in dogs [33] resulted in elevations in ocular temperature. The higher temperature of the eye caruncle herein found could be related to ocular vasodilatation causing the greater heat dissipation identified here by the thermographic measurements. Vasodilation can be due to fear-induced activation of the parasympathetic nervous system, in this case by the facial nerve (VII pair) through the pterygopalatine ganglion and the subsequent postganglionic fibers [51]. The eye and its surrounding skin tissue provide an image that may reflect the sympathetic/parasympathetic balance of the animal [52]. Furthermore, when a “fight/flight” reaction is triggered [42], there is an increase in core body temperature [53], primarily observed as a transient acute response, lasting less than one hour [31]. Given that cortisol induces various thermogenic processes in tissue metabolism, such as gluconeogenesis, glycolysis, proteolysis, and lipolysis [54,55], it is possible to suggest that alterations in eye temperature could be influenced, to some extent, by the activation of the hypothalamic–pituitary–adrenal (HPA) axis and the visceral nervous system in response to stimulation [55]. On the contrary, other studies showed that the application of a painful stimulus did not result in any observable alterations in the ocular temperature of sheep [56] or in some instances observed a rapid decrease in eye temperature following exposure to a stressor, followed by a subsequent increase back to baseline values [28,30,57]. This decrease is believed to be followed by thermogenic reactions in tissue metabolism, which are induced by glucocorticoids and may be responsible for the subsequent increase in temperature [54]. The reasons behind the fluctuation of ocular temperature in response to stress remain uncertain. It is possible that variations in data collection methods, such as the timing of sampling, could contribute to the observed discrepancies among studies [45]. Additionally, it would be interesting to explore whether the level of the stimulus could be a factor in the absence of an emotional response.

In terms of behavioral reaction, our findings showed a significant increase in the time spent with ears forward in the positive group, awaiting a food reward, while sheep in the negative group spent more time with their ears backwards waiting for the opening of the umbrella. The presence of the “accompanying sheep” reduced the potential for stress induced by separation from the flock, and the gradual habituation to the experimental set up allowed for the assessment of behavioral reactions without the influence of environmental novelty. Several studies in different animal species [37,58,59,60,61] have investigated the possible role of ear postures in social communication and the expression of the internal state [37,58,59,60,61]. For example, in dairy cows, a relaxed ear posture is indicative of a positive, low-arousal emotional state [62], and, in horses and pigs, a negative emotional experience might be associated with backwards ears [63,64]. Ruminants possess well-developed muscles around their ears, allowing them a high degree of independent movement in both the forward and backward directions [36]; however, ear postures are highly species-dependent [62], and, for instance, both backwards and forwards ears might be associated either with positive or negative situations [35,36,62,65]. In sheep, Reefmann and colleagues [35,36] found that regarding the number of ear posture changes, forward and asymmetric ear postures were highest during negative experiences of social isolation, while a “passive” ear posture were observed during positive ones. Boissy et al. (2011) [37] found similar results; in their study, sheep were exposed to situations of varying degrees of suddenness and familiarity, negative contrast, and controllability [37]. Asymmetrical ear posture was mainly displayed in response to sudden situations in relation with a startle response (surprise). During the anticipation phase, this is reduced by the effects of the conditioning. In our study, sheep in the positive group kept their ears forward more often, since the experience was of a pleasant nature. However, these results are in contrast with the findings of the mentioned studies [35,36,37]. Reefmann and colleagues [35,36] focused their conclusions mainly on the frequency of ear posture changes, rather than on the duration, in positive–negative contrast paradigms [35,36]. Positive–negative contrasts pertain to the phenomenon observed when an animal undergoes conditioning to anticipate a specific reward or event, and, subsequently, that reward or event is altered to be of either diminished perceived value or heightened perceived value [66]. Moreover, there is a strong interrelation of attention and emotion [67], suggesting that the scrutiny of ear postures remains unaffected by attention, emphasizing that attention forms an integral component of an emotional response. The level of attention is increased during the anticipation of feeding as a positive event [68], enabling prompt detection of the occurrence or absence of a reward. The certainty of anticipation of positive events might be perceived as a controllable situation, as displayed by the raised ear posture reported by Boissy and colleagues (2011) [37]. Moreover, the increased level of activity when anticipating the upcoming event was reported as a result of the learnt association between the occurrence of a neutral sound and the oncoming food reward [69]. This could explain the increased time spent in the forward position during the anticipation phase in the positive group, whereas the successful acquisition of these rewards induces positive affective states [70]; however, further studies are needed to investigate and clarify how the positive and negative value of an experience affects active behavior [69].

In our study, cortisol levels increased, although not significantly, in both positive and negative groups post-treatment. The anticipation of positive events could potentially induce transitioning stress and frustration if the reward is bestowed belatedly [36]. We can also hypothesize that the certain anticipation of a positive experience, such as waiting for a food reward, can also make the context stimulating where the sheep actively direct their attention to the surroundings, as observed for dairy cows [61].

Combining lacrimal caruncle temperature, cortisol levels, and ear position in sheep with positive and negative expectations can be considered an innovative approach for studying the emotional reactions of sheep. Additional research is required to further examine the relationship between these parameters in order to gain more comprehensive insights into the optimal timing for sample collection. It is important to consider that the present study included a small sample size and was run in an experimental situation; therefore, some attention should be paid when we want to generalize the results to on-farm situations.

## 5. Conclusions

Our results confirmed that infrared thermography could be applied as a non-invasive tool to identify negative and positive events that may elicit emotional states affecting physiological response in sheep since the thermal, physiological, and behavioral facial response were influenced by negative events in sheep. Therefore, such evidence shows that the use of IRT might be able to assess emotional response in this species. Further research is needed to investigate, in more depth, the correlation between eye temperature, cortisol-level variations, and behavioral reactions to gather information about the best timing for collecting samples, thus improving the reliability and applicability of this technology in on-farm situations.

## Figures and Tables

**Table 1:** Summary representation of the test phases for both of the treatment groups.

Positive Group	Negative Group
Baseline (10 s)	Baseline (10 s)
Sound (5 s)	Sound (5 s)
Anticipation (10 s)	Anticipation (10 s)
Feed distribution (5 s)	Umbrella opening (5 s)
Consumption phase (10 s)	Sheep reaction (10 s)

## Data Availability

The data presented in this study are available upon request from the corresponding author E.D.C. The data are not publicly available due to privacy restrictions.

## References

[1] Chincarini M., Dalla Costa E., Qiu L., Spinelli L., Cannas S., Palestrini C., Canali E., Minero M., Cozzi B., Ferri N. (2020). Reliability of FNIRS for Noninvasive Monitoring of Brain Function and Emotion in Sheep. Sci. Rep..

[2] Hemsworth P., Mellor D., Cronin G., Tilbrook A. (2015). Scientific Assessment of Animal Welfare. N. Z. Vet. J..

[3] Ekman P. (1982). Emotion in the Human Face.

[4] Fox E. (2008). Emotion Science Cognitive and Neuroscientific Approaches to Understanding Human Emotions.

[5] Boissy A., Manteuffel G., Jensen M.B., Moe R.O., Spruijt B., Keeling L.J., Winckler C., Forkman B., Dimitrov I., Langbein J. (2007). Assessment of Positive Emotions in Animals to Improve Their Welfare. Physiol. Behav..

[6] Panksepp J., Eckman P., Davidson R.J. (1994). Evolution Constructed the Potential for Subjective Experience within the Neurodynamics of the Mammalian Brain. The Nature of Emotion: Fundamental Questions.

[7] Rolls E. (2000). Precis of the Brain and Emotion. Behav. Brain Sci..

[8] Jarvis E.D., Güntürkün O., Bruce L., Csillag A., Karten H., Kuenzel W., Medina L., Paxinos G., Perkel D.J., Shimizu T. (2005). Avian Brains and a New Understanding of Vertebrate Brain Evolution. Nat. Rev. Neurosci..

[9] Reiner A., Perkel D.J., Bruce L.L., Butler A.B., Csillag A., Kuenzel W., Medina L., Paxinos G., Shimizu T., Striedter G. (2004). Revised Nomenclature for Avian Telencephalon and Some Related Brainstem Nuclei. J. Comp. Neurol..

[10] Tooby J., Cosmides L. (1990). The Past Explains the Present. Ethol. Sociobiol..

[11] Levenson R.W., Ekman P., Davidson R.J. (1994). Human Emotion: A Functionalist View. The Nature of Emotion: Fundamental Questions.

[12] Lazarus R.S. (1991). Cognition and Motivation in Emotion. Am. Psychol..

[13] Fredrickson B.L. (1998). Cultivated Emotions: Parental Socialization of Positive Emotions and Self-Conscious Emotions. Psychol. Inq..

[14] Fredrickson B.L. (2001). The Role of Positive Emotions in Positive Psychology: The Broaden-and-Build Theory of Positive Emotions. Am. Psychol..

[15] Broom D. (1998). Welfare, Stress, and Evolution of Feelings. Adv. Study Behav..

[16] Neethirajan S., Reimert I., Kemp B. (2021). Measuring Farm Animal Emotions—Sensor-Based Approaches. Sensors.

[17] Lezama-García K., Orihuela A., Olmos-Hernández A., Reyes-Long S., Mota-Rojas D. (2019). Facial Expressions and Emotions in Domestic Animals. CABI Rev..

[18] Whittaker A.L., Marsh L.E. (2019). The Role of Behavioural Assessment in Determining “positive” Affective States in Animals. CABI Rev..

[19] Cannas S., Palestrini C., Canali E., Cozzi B., Ferri N., Heinzl E., Minero M., Chincarini M., Vignola G., Dalla Costa E. (2018). Thermography as a Non-Invasive Measure of Stress and Fear of Humans in Sheep. Animals.

[20] Dantzer R. (1988). Les Émotions.

[21] Mason J.W. (1972). A Re-Evaluation of the Concept of “Non-Specificity” in Stress Theory. Principles, Practices, and Positions in Neuropsychiatric Research.

[22] Colborn D.R., Thompson D.L., Roth T.L., Capehart J.S., White K.L. (1991). Responses of Cortisol and Prolactin to Sexual Excitement and Stress in Stallions and Geldings. J. Anim. Sci..

[23] Borgs P., Mallard B.A. (1998). Immune-Endocrine Interactions in Agricultural Species: Chromium and Its Effect on Health and Performance. Domest. Anim. Endocrinol..

[24] Mormède P., Foury A., Terenina E., Knap P.W. (2011). Breeding for Robustness: The Role of Cortisol. Animal.

[25] Kammersgaard T.S., Malmkvist J., Pedersen L.J. (2013). Infrared Thermography—A Non-Invasive Tool to Evaluate Thermal Status of Neonatal Pigs Based on Surface Temperature. Animal.

[26] Travain T., Valsecchi P. (2021). Infrared Thermography in the Study of Animals’ Emotional Responses: A Critical Review. Animals.

[27] Stewart M., Schaefer A.L., Haley D.B., Colyn J., Cook N.J., Stafford K.J., Webster J.R. (2008). Infrared Thermography as a Non-Invasive Method for Detecting Fear-Related Responses of Cattle to Handling Procedures. Anim. Welf..

[28] Stewart M., Webster J.R., Verkerk G.A., Schaefer A.L., Colyn J.J., Stafford K.J. (2007). Non-Invasive Measurement of Stress in Dairy Cows Using Infrared Thermography. Physiol. Behav..

[29] Stewart M., Wilson M.T., Schaefer A.L., Huddart F., Sutherland M.A. (2017). The Use of Infrared Thermography and Accelerometers for Remote Monitoring of Dairy Cow Health and Welfare. J. Dairy Sci..

[30] Stewart M., Stafford K.J.J., Dowling S.K.K., Schaefer A.L.L., Webster J.R.R. (2008). Eye Temperature and Heart Rate Variability of Calves Disbudded with or without Local Anaesthetic. Physiol. Behav..

[31] Adriaan Bouwknecht J., Olivier B., Paylor R.E. (2007). The Stress-Induced Hyperthermia Paradigm as a Physiological Animal Model for Anxiety: A Review of Pharmacological and Genetic Studies in the Mouse. Neurosci. Biobehav. Rev..

[32] Hernández-Avalos I., Mota-Rojas D., Mendoza-Flores J.E., Casas-Alvarado A., Flores-Padilla K., Miranda-Cortes A.E., Torres-Bernal F., Gómez-Prado J., Mora-Medina P. (2021). Nociceptive Pain and Anxiety in Equines: Physiological and Behavioral Alterations. Vet. World.

[33] Travain T., Colombo E.S., Heinzl E., Bellucci D., Prato Previde E., Valsecchi P. (2015). Hot Dogs: Thermography in the Assessment of Stress in Dogs (*Canis familiaris*)—A Pilot Study. J. Vet. Behav..

[34] Hall C., Kay R., Yarnell K. (2014). Assessing Ridden Horse Behavior: Professional Judgment and Physiological Measures. J. Vet. Behav..

[35] Reefmann N., Wechsler B., Gygax L. (2009). Behavioural and Physiological Assessment of Positive and Negative Emotion in Sheep. Anim. Behav..

[36] Reefmann N., Bütikofer Kaszàs F., Wechsler B., Gygax L. (2009). Ear and Tail Postures as Indicators of Emotional Valence in Sheep. Appl. Anim. Behav. Sci..

[37] Boissy A., Aubert A., Désiré L., Greiveldinger L., Delval E., Veissier I., Desire L. (2011). Cognitive Sciences to Relate Ear Postures to Emotions in Sheep. Anim. Welf..

[38] Chapagain D., Uvnäs-Moberg K., Lidfors L.M. (2014). Investigating the Motivation to Play in Lambs. Appl. Anim. Behav. Sci..

[39] Vögeli S., Wechsler B., Gygax L. (2014). Welfare by the Ear: Comparing Relative Durations and Frequencies of Ear Postures by Using an Automated Tracking System in Sheep. Anim. Welf..

[40] Mallick S.P., Zickler T.E., Kriegman D.J., Belhumeur P.N. (2005). Beyond Lambert: Reconstructing Specular Surfaces Using Color. Proc. IEEE Comput. Soc. Conf. Comput. Vis. Pattern Recognit..

[41] Bartolomé E., Sánchez M.J.J., Molina A., Schaefer A.L.L., Cervantes I., Valera M. (2013). Using Eye Temperature and Heart Rate for Stress Assessment in Young Horses Competing in Jumping Competitions and Its Possible Influence on Sport Performance. Animal.

[42] Dai F., Cogi N.H., Heinzl E.U.L., Dalla Costa E., Canali E., Minero M. (2015). Validation of a Fear Test in Sport Horses Using Infrared Thermography. J. Vet. Behav. Clin. Appl. Res..

[43] Grayess (2007). IRT Analyser Users’ Manual.

[44] Bonelli F., Rota A., Aurich C., Ille N., Camillo F., Panzani D., Sgorbini M. (2019). Determination of Salivary Cortisol in Donkey Stallions. J. Equine Vet. Sci..

[45] Arfuso F., Acri G., Piccione G., Sansotta C., Fazio F., Giudice E., Giannetto C. (2022). Eye Surface Infrared Thermography Usefulness as a Noninvasive Method of Measuring Stress Response in Sheep during Shearing: Correlations with Serum Cortisol and Rectal Temperature Values. Physiol. Behav..

[46] Stewart M., Verkerk G.A., Stafford K.J., Schaefer A.L., Webster J.R. (2010). Noninvasive Assessment of Autonomic Activity for Evaluation of Pain in Calves, Using Surgical Castration as a Model. J. Dairy Sci..

[47] Lei M.C., Félix L., Cardoso R., Monteiro S.M., Silva S., Venâncio C. (2023). Non-Invasive Biomarkers in Saliva and Eye Infrared Thermography to Assess the Stress Response of Calves during Transport. Animals.

[48] Uddin J., McNeill D.M., Phillips C.J.C. (2023). Measuring Emotions in Dairy Cows: Relationships between Infrared Temperature of Key Body Parts, Lateralised Behaviour and Milk Production. Appl. Anim. Behav. Sci..

[49] Yarnell K., Hall C., Billett E. (2013). An Assessment of the Aversive Nature of an Animal Management Procedure (Clipping) Using Behavioral and Physiological Measures. Physiol. Behav..

[50] Fenner K., Yoon S., White P., Starling M., McGreevy P. (2016). The Effect of Noseband Tightening on Horses’ Behavior, Eye Temperature, and Cardiac Responses. PLoS ONE.

[51] Cozzi B., Granato A., Merighi A. (2017). Neuroanatomia Dell’uomo.

[52] Sutherland M.A., Worth G.M., Dowling S.K., Lowe G.L., Cave V.M., Stewart M. (2020). Evaluation of Infrared Thermography as a Non-Invasive Method of Measuring the Autonomic Nervous Response in Sheep. PLoS ONE.

[53] Oka T., Oka K., Hori T. (2001). Mechanisms and Mediators of Psychological Stress-Induced Rise in Core Temperature. Psychosom. Med..

[54] Sapolsky R.M., Romero L.M., Munck A.U. (2000). How Do Glucocorticoids Influence Stress Responses? Integrating Permissive, Suppressive, Stimulatory, and Preparative Actions. Endocr. Rev..

[55] Valera M., Bartolomé E., Sánchez M.J., Molina A., Cook N., Schaefer A. (2012). Changes in Eye Temperature and Stress Assessment in Horses During Show Jumping Competitions. J. Equine Vet. Sci..

[56] Stubsjøen S.M., Flø A.S., Moe R.O., Janczak A.M., Skjerve E., Valle P.S., Zanella A.J. (2009). Exploring Non-Invasive Methods to Assess Pain in Sheep. Physiol. Behav..

[57] Church J.S., Hegadoren P.R., Paetkau M.J., Miller C.C., Regev-Shoshani G., Schaefer A.L., Schwartzkopf-Genswein K.S. (2014). Influence of Environmental Factors on Infrared Eye Temperature Measurements in Cattle. Res. Vet. Sci..

[58] Schmied C., Waiblinger S., Scharl T., Leisch F., Boivin X. (2008). Stroking of Different Body Regions by a Human: Effects on Behaviour and Heart Rate of Dairy Cows. Appl. Anim. Behav. Sci..

[59] Wathan J., McComb K. (2014). The Eyes and Ears Are Visual Indicators of Attention in Domestic Horses. Curr. Biol..

[60] Bellegarde L.G.A., Haskell M.J., Duvaux-Ponter C., Weiss A., Boissy A., Erhard H.W. (2017). Face-Based Perception of Emotions in Dairy Goats. Appl. Anim. Behav. Sci..

[61] Battini M., Agostini A., Mattiello S. (2019). Understanding Cows’ Emotions on Farm: Are Eye White and Ear Posture Reliable Indicators?. Animals.

[62] Proctor H.S., Carder G. (2014). Can Ear Postures Reliably Measure the Positive Emotional State of Cows?. Appl. Anim. Behav. Sci..

[63] Reimert I., Bolhuis J.E., Kemp B., Rodenburg T.B. (2013). Indicators of Positive and Negative Emotions and Emotional Contagion in Pigs. Physiol. Behav..

[64] Dalla Costa E., Pascuzzo R., Leach M.C., Dai F., Lebelt D., Vantini S., Minero M. (2018). Can Grimace Scales Estimate the Pain Status in Horses and Mice? A Statistical Approach to Identify a Classifier. PLoS ONE.

[65] Edgar J.L., Nicol C.J., Pugh C.A., Paul E.S. (2013). Surface Temperature Changes in Response to Handling in Domestic Chickens. Physiol. Behav..

[66] Flaherty C.F., Rowan G.A. (1986). Successive, Simultaneous, and Anticipatory Contrast in the Consumption of Saccharin Solutions. J. Exp. Psychol. Anim. Behav. Process..

[67] Carretié L., Mercado F., Tapia M., Hinojosa J.A. (2001). Emotion, Attention, and the ‘Negativity Bias’, Studied through Event-Related Potentials. Int. J. Psychophysiol..

[68] Melges F.T., Poppen R.L. (1976). Expectation of Rewards and Emotional Behavior in Monkeys. J. Psychiatr. Res..

[69] Chincarini M., Qiu L., Spinelli L., Torricelli A., Minero M., Dalla Costa E., Mariscoli M., Ferri N., Giammarco M., Vignola G. (2018). Evaluation of Sheep Anticipatory Response to a Food Reward by Means of Functional Near-Infrared Spectroscopy. Animals.

[70] Burman O., McGowan R., Mendl M., Norling Y., Paul E., Rehn T., Keeling L. (2011). Using Judgement Bias to Measure Positive Affective State in Dogs. Appl. Anim. Behav. Sci..

